# Efficacy of osimertinib in epidermal growth factor receptor-mutated non-small-cell lung cancer patients with pleural effusion

**DOI:** 10.1186/s12885-022-09701-2

**Published:** 2022-06-01

**Authors:** Hiroshi Nokihara, Hirokazu Ogino, Atsushi Mitsuhashi, Kensuke Kondo, Ei Ogawa, Ryohiko Ozaki, Yohei Yabuki, Hiroto Yoneda, Kenji Otsuka, Yasuhiko Nishioka

**Affiliations:** 1 Department of Respiratory Medicine and Rheumatology, Graduate School of Biomedical Sciences, Tokushima University, 3-18-15, Kuramoto-cho, Tokushima, 770-8503 Japan; 2 Present Address: Respiratory Medicine, Center Hospital of the National Center for Global Health and Medicine, 1-21-1 Toyama, Shinjuku-ku, Tokyo, 162-8655 Japan

**Keywords:** Non-small cell lung cancer, Epidermal growth factor receptor, Osimertinib, Pleural effusion

## Abstract

**Background:**

Osimertinib is a standard first-line treatment for advanced non-small-cell lung cancer (NSCLC) harboring *epidermal growth factor receptor (EGFR)* mutations. Although malignant pleural effusion (PE) is a common clinical problem in NSCLC, information about the efficacy of osimertinib in patients with PE is limited, especially regarding its efficacy in *EGFR* T790M-negative patients with PE remains unclear.

**Methods:**

We retrospectively reviewed the medical records of patients with NSCLC harboring *EGFR* mutations who were treated with osimertinib in our institution between May 2016 and December 2020.

**Results:**

A total of 63 patients with *EGFR* mutated NSCLC were treated with osimertinib; 33 (12 with PE) had no *EGFR* T790M mutation, while 30 (12 with PE) had *EGFR* T790M mutation. In *EGFR* T790M-negative NSCLC, the progression-free survival (PFS) of the patients with PE was comparable to that of the patients without PE (median PFS 19.8 vs. 19.8 months, *p* = 0.693). In *EGFR* T790M- positive NSCLC, the PFS and overall survival (OS) of the patients with PE were significantly shorter than those of the patients without PE (median PFS 16.8 vs. 8.3 months, *p* = 0.003; median OS 44.9 vs. 14.2 months, *p* = 0.007). In the multivariate analysis, the presence of PE was independently associated with shorter PFS and OS in *EGFR* T790M-positive NSCLC patients, but not *EGFR* T790M-negative patients.

**Conclusions:**

These data suggest the efficacy of osimertinib may differ between *EGFR* T790M-positive and -negative NSCLC patients with PE.

**Supplementary Information:**

The online version contains supplementary material available at 10.1186/s12885-022-09701-2.

## Introduction

Malignant pleural effusion (PE) is a common clinical problem in non-small-cell lung cancer (NSCLC). Previous studies have reported that malignant PE is present in 15% to 20% of patients with NSCLC, and that it is associated with a poor prognosis in patients with advanced NSCLC [1–3]. Even minimal PE (defined as thickness < 10 mm on chest computed tomography [CT] scan) is an independent prognostic factor of a worse survival among patients with NSCLC, and the survival of patients with minimal PE is as short as that of patients with malignant PE in stage IV disease [3].

The introduction of epidermal growth factor receptor (EGFR)-tyrosine kinase inhibitors (TKIs) into the treatment paradigm of NSCLC harboring *EGFR* mutations dramatically improved clinical outcomes. For advanced NSCLC patients with *EGFR*-activating mutations involving deletions in exon 19 (exon 19 deletion) or a substitution mutation in exon 21, specifically Leu858Arg (L858R), EGFR-TKIs are the standard first-line therapies. The presence of *EGFR* mutations has been reported to be significantly associated with PE and may play an important role in the formation of malignant PE [4, 5]. The studies examining *EGFR*-mutated NSCLC patients treated with erlotinib, first-generation EGFR-TKIs, have shown that the presence of malignant PE is associated with a shorter progression-free survival (PFS) and overall survival (OS) [6, 7].

Osimertinib is a third-generation EGFR-TKI that potently and selectively inhibits both EGFR-TKI-sensitizing and T790M-resistant mutations. The phase III study (AURA 3) showed that osimertinib improved PFS over platinum combined chemotherapy in patients with *EGFR* T790M-positive NSCLC whose disease had progressed after EGFR-TKI treatment [8, 9]. In Japan, osimertinib has been approved for NSCLC harboring an *EGFR* Thr790Met mutation in exon 20 (T790M mutation) since 2016. The phase III study (FLAURA) found that osimertinib led to a significant improvement in the PFS and OS over first-generation EGFR-TKIs in untreated advanced NSCLC patients with *EGFR* mutation [10, 11]. Since then, osimertinib has become a standard first-line treatment for advanced NSCLC harboring *EGFR* mutations.

A few retrospective studies have investigated the efficacy of osimertinib in patients with *EGFR* T790M-positive NSCLC with PE [12–14]. Patients with PE were reported to have a significantly shorter median time to treatment failure (TTF) than those without PE, as well as a shorter median OS [12]. Conversely, it has been shown that the median PFS with osimertinib treatment did not significantly differ between patients with and without PE [13, 14]. Therefore, the efficacy of osimertinib in *EGFR* T790M-positive patients with PE remains unclear.

Recently, osimertinib is usually administered as a first-line treatment to patients with *EGFR* T790M-negative NSCLC, and the efficacy of osimertinib in *EGFR* T790M-negative patients with PE is unknown. Thus, we conducted a retrospective study to investigate the efficacy of osimertinib in the treatment of *EGFR* T790M-negative NSCLC patients with PE.

## Methods

### Patients

We retrospectively reviewed the medical records of all patients, who were diagnosed with NSCLC harboring *EGFR* mutation and who were treated with osimertinib in Tokushima University Hospital between May 2016 and December 2020. The end of the follow-up period was June 30, 2021.

This study was performed in accordance with the Declaration of Helsinki and was approved by the institutional review board.

### Assessments

We used either an Oncomine Dx Target Test or Cobas *EGFR* Mutation Test version 2 [15] for the *EGFR* mutations analyses of tissue or cytology samples at the time of the diagnosis. After treatment with first- or second-generation EGFR-TKIs, patients were confirmed to have the *EGFR* T790M mutation in tissue, cytology or blood samples using the Cobas *EGFR* Mutation Test kit version 2. These tests were performed at SRL, Inc. (Tokyo, Japan) in a clinical practice setting.

Patients with positive pleural fluid cytology results, pleural effusion requiring drainage, or presenting with multiple pleural nodules and nodular pleural thickening with pleural fluid on a CT scan was diagnosed as having PE. According to the thickness of the pleural fluid (judged relative to a criterion of 10 mm on chest CT scans), patients with PE were classified into two groups: minimal PE (thickness < 10 mm) and malignant PE [3]. We diagnosed the presence of PE before beginning with osimertinib treatment.

The tumor response to osimertinib was categorized as either complete response (CR), partial response (PR), stable disease (SD), progressive disease (PD), or not evaluated (NE), according to the Response Evaluation Criteria in Solid Tumors (RECIST) version 1.1 [16]. PFS was defined as the period from the start of treatment with osimertinib to the date of clinical or radiographic disease progression or death from any cause, and in the absence of confirmation of disease progression or death data were censored at the last date the patient was known to be alive. OS was defined as the period from the commencement of osimertinib treatment to the date of death from any cause, and in the absence of confirmation of death data were censored at the last date the patient was known to be alive.

### Statistical analyses

Baseline characteristics were compared between patients with and without PE using the Mann-Whitney *U* test or Fisher’s exact test for categorical variables. The PFS and OS were estimated by the Kaplan-Meier method, and their statistical differences were analyzed by the log-rank test. Univariate and multivariate analyses were performed using Cox proportional hazards regression models. In these analyses, *p*-values of < 0.05 were considered to indicate a statistically significant difference. The statistical analyses were performed using EZR version 1.54 (Saitama Medical Center, Jichi Medical University, Saitama, Japan) [17].

## Results

### Patient disposition and characteristics

A total of 63 patients with *EGFR* mutated NSCLC who were treated with osimertinib were identified (Additional file 1: Figure A1). Among 63 patients, 33 patients had no *EGFR* T790M mutation (with PE, *n* = 12; maximum thickness < 10 mm, *n* = 4; maximum thickness ≥ 10 mm, *n* = 8; cytologically confirmed malignant cells, *n* = 6). Thirty patients had *EGFR* T790M mutation (with PE, *n* = 12; maximum thickness < 10 mm, *n* = 7; maximum thickness ≥ 10 mm, *n* = 5; cytologically confirmed malignant cells, *n* = 3) including a patient with both L858R and T790M (de novo) at the time of diagnosis. The baseline characteristics are shown in Table 1. Among 33 *EGFR* T790M-negative patients, one patient relapsed after curative chemoradiotherapy, three relapsed after adjuvant chemotherapy, and *EGFR* mutations was confirmed in one patient after the start of platinum-based chemotherapy. One patient discontinued first-generation EGFR-TKI treatment because of adverse events, and received osimertinib without confirmation of the *EGFR* T790M mutation status. The baseline characteristics of the patients with and without PE did not differ to a statistically significant extent in *EGFR* T790M-positive or negative patients.

**Table 1:** Patient characteristics at baseline

	T790M negative (*n* = 33)					T790M positive (*n* = 30)				
	With PE (*n* = 12)		Without PE (*n* = 21)		*p*-Value	With PE (*n* = 12)		Without PE (*n* = 18)		*p*-Value
Median age (range), years	72	(48–88)	70	(38–81)	0.587	68.5	(28–83)	70	(44–86)	0.433
Gender, n (%)										
Male	4	(33.3%)	6	(28.6%)		3	(25.0%)	8	(44.4%)	
Female	8	(66.7%)	15	(71.4%)	1.000	9	(75.0%)	10	(55.6%)	0.442
Smoking history, n (%)										
Never	9	(75.0%)	15	(71.4%)		9	(75.0%)	10	(55.6%)	
Former/Current	3	(25.0%)	6	(28.6%)	1.000	3	(25.0%)	8	(44.4%)	0.442
ECOG performance status, n (%)										
0–1	10	(83.3%)	20	(95.2%)		8	(66.7%)	15	(83.3%)	
2–3	2	(16.7%)	1	(4.8%)	0.538	4	(33.3%)	3	(16.7%)	0.392
Histology, n (%)										
Adenocarcinoma	11	(91.7%)	19	(90.5%)		12	(100.0%)	18	(100.0%)	
Squamous cell carcinoma	1	(8.3%)	0	(0.0%)		0	(0.0%)	0	(0.0%)	
Other	0	(0.0%)	2	(9.5%)	0.279	0	(0.0%)	0	(0.0%)	NA
Clinical stage, n (%)										
III	0	(0.0%)	1	(4.8%)		0	(0.0%)	0	(0.0%)	
IV	12	(100.0%)	15	(71.4%)		10	(83.3%)	15	(83.3%)	
Recurrence	0	(0.0%)	5	(23.8%)	0.133	2	(16.7%)	3	(16.7%)	1.000
Metastases, n (%)										
Brain	7	(58.3%)	11	(52.4%)	1.000	6	(50.0%)	12	(66.7%)	0.458
Bone	4	(33.3%)	11	(52.4%)	0.469	6	(50.0%)	3	(16.7%)	0.102
Liver	2	(16.7%)	1	(4.8%)	0.538	3	(25.0%)	1	(5.6%)	0.274
*EGFR* mutation status, n (%)										
Exon 19 deletion	7	(58.3%)	10	(47.6%)		6	(50.0%)	7	(38.9%)	
L858R	5	(41.7%)	10	(47.6%)		5	(41.7%)	11	(61.1%)	
Other	0	(0.0%)	1	(4.8%)	0.825	1	(8.3%)	0	(0.0%)	0.351
Prior anticancer drug treatment, n (%)										
None	11	(91.7%)	19	(90.5%)	1.000	0	(0.0%)	0	(0.0%)	NA
EGFR-TKI	0	(0.0%)	1	(4.8%)	1.000	12	(100.0%)	18	(100.0%)	NA
Chemotherapy	3	(25.0%)	2	(9.5%)	0.233	7	(58,3%)	9	(55.6%)	0.722

*PE* pleural effusion, *ECOG* Eastern Cooperative Oncology Group, *EGFR* epidermal growth factor receptor, *TKI* tyrosine kinase inhibitor, *NA* not applicable

### Efficacy of osimertinib in the patients with PE

The objective response rate (ORR) in the patients with PE was lower than that in the patients without PE in both *EGFR* T790M-negative (58.3% vs. 71.4%, *p* = 0.443) and in *EGFR* T790M-positive (66.7% vs. 83.3%, *p* = 0.290) patients, although the result did not reach statistical significance (Table 2). *EGFR* T790M-negative patients with and without PE showed a similar disease control rate (DCR) (91.7% vs. 95.2%) (Table 2).

**Table 2:** Overall responses

	T790M negative (*n* = 33)					T790M positive (*n* = 30)				
	With PE (*n* = 12)		Without PE (*n* = 21)		*p*-Value	With PE (*n* = 12)		Without PE (*n* = 18)		*p*-Value
CR	0	(0.0%)	0	(0.0%)		0	(0.0%)	0	(0.0%)	
PR	7	(58.3%)	15	(71.4%)		8	(66.7%)	15	(83.3%)	
SD	4	(33.3%)	5	(23.8%)		4	(33.3%)	3	(16.7%)	
PD	1	(8.3%)	0	(0.0%)		0	(0.0%)	0	(0.0%)	
NE	0	(0.0%)	1	(4.8%)		0	(0.0%)	0	(0.0%)	
ORR		58.3%		71.4%	0.443		66.7%		83.3%	0.290
DCR		91.7%		95.2%	0.679		100.0%		100.0%	NA

*CR* complete response, *PR* partial response, *SD* stable disease, *PD* progressive disease, *NE* not evaluated, *ORR* objective response rate, *DCR* disease control rate, *NA* not applicable

**Fig. 1:**
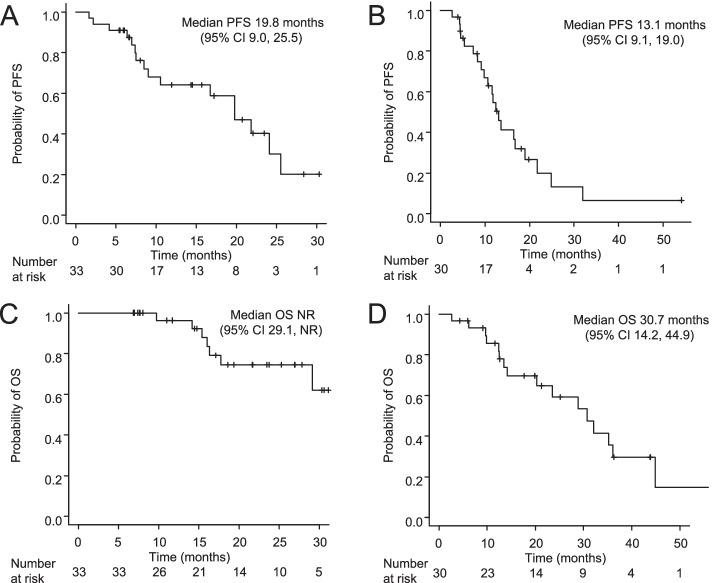
Kaplan–Meier curve of progression-free survival (**A**, **B**) and overall survival (**C**, **D**) in *EGFR* T790M-negative (**A**, **C**) and *EGFR* T790M-posittive patients (**B**, **D**). PFS, progression-free survival; OS, overall survival; NR, not reached; CI, confidence interval

**Fig. 2:**
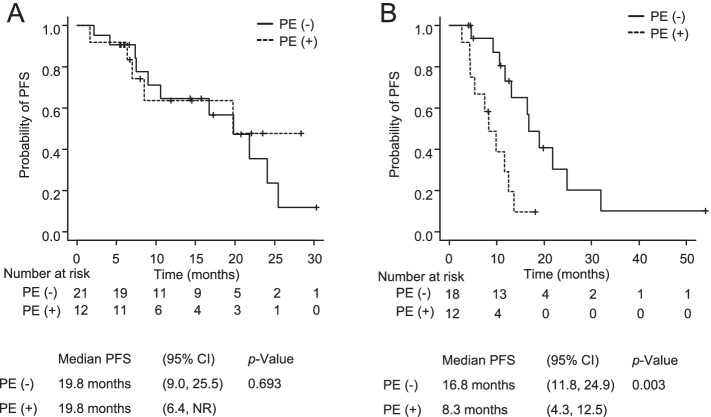
Kaplan–Meier curve of progression-free survival in *EGFR* T790M-negative (**A**) and *EGFR* T790M-positive patients (**B**) according to the presence or absence of pleural effusion. PE, pleural effusion; PFS, progression-free survival; NR, not reached; CI, confidence interval

**Fig. 3:**
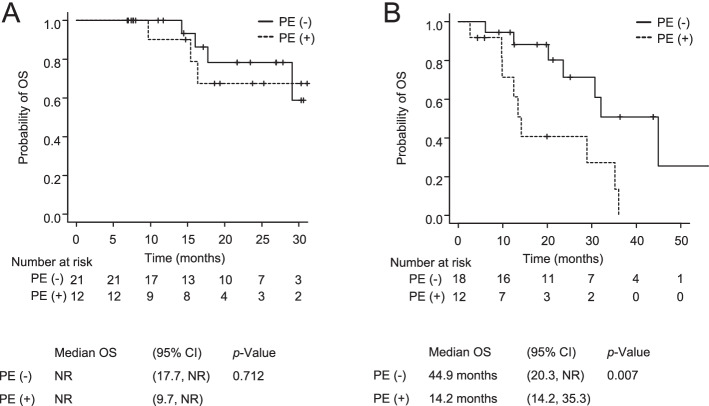
Kaplan–Meier curve of overall survival in *EGFR* T790M-negative (**A**) or *EGFR* T790M-positive patients (**B**) according to the presence or absence of pleural effusion. PE, pleural effusion; OS, overall survival; NR, not reached; CI, confidence interval

The median follow-up period was 17.1 months (range, 6.9 to 31.1 months) for all *EGFR* T790M-negative patients and 19.8 months (range, 2.6 to 56.6 months) for all *EGFR* T790M-positive patients. The median PFS and OS were 19.8 months (95% confidence interval [CI] 9.0 to 25.5) and not reached (NR) (95%CI 29.1 to NR), respectively, in *EGFR* T790M-negative patients, and 13.1 months (95%CI 9.1 to 19.0) and 30.7 months (95% CI 14.2 to 44.9) in *EGFR*-T790M positive patients (Fig. 1). In *EGFR*-T790M negative NSCLC, the PFS and OS of the patients with PE were comparable to those of the patients without PE (median PFS 19.8 vs. 19.8 months, *p* = 0.693; median OS NR vs. NR, *p* = 0.712) (Figs. 2A and 3A). In *EGFR* T790M-positive NSCLC, the PFS and OS of the patients with PE were significantly shorter in comparison to the patients without PE (median PFS 16.8 vs. 8.3 months, *p* = 0.003; median OS 44.9 vs. 14.2 months, *p* = 0.007) (Figs. 2B, 3B). In *EGFR*-mutated patients with PE, the PFS and OS did not significantly differ between the patients with exon 19 deletion and those with L858R (median PFS NR vs. 8.3 months, *p* = 0.056; median OS 28.9 vs. 25.8 months, *p* = 0.777).

The multivariate analysis showed that the presence of PE was significantly and independently associated with shorter PFS (hazard ratio [HR] 7.31, 95% CI 2.05 to 26.03, *P* = 0.002) and OS (HR 4.11, 95% CI 1.08 to 15.62, *P* = 0.038) in *EGFR* T790M-positive patients treated with osimertinib, while in *EGFR* T790M-negative patients, the presence of PE did not significantly influence PFS or OS (Table 3). Only 3 of the 33 *EGFR* T790M-negative patients had a performance status of 2 or 3; these patients had no disease progression and were alive at the end of the follow-up period. As is well known, in *EGFR* T790M-negative patients, *EGFR* mutation (L858R or other) was significantly associated with shorter PFS (hazard ratio [HR] 5.90, 95% CI 1.22 to 28.54, *P* = 0.027) (Table 3).

**Table 3:** Cox proportional hazard regression analyses

	Univariate analysis			Multivariate analysis		
Variables	HR (95% CI)		*P* value	HR (95% CI)		*P* value
T790M negative patients						
PFS						
Age (≥ 75/ < 74)	0.52	(0.16, 1.68)	0.276	1.13	(0.18, 6.97)	0.897
Gender (male/female)	1.84	(0.64, 5.32)	0.260	2.92	(0.57, 14.95)	0.198
ECOG performance status (2-3/0-1)	4,18 × 10^–9^	(0.00, INF)	0.998	7.19 × 10^–10^	(0.00, INF)	0.998
*EGFR* mutation status (L858R or other/exon 19 deletion)	2.20	(0.80, 6.02)	0.126	5.90	(1.22, 28.54)	0.027
PE ( +/-)	0.81	(0.28, 2.33)	0.694	1.89	(0.38, 9.34)	0.434
Brain metastases (+/-)	0.87	(0.13, 2.41)	0.787	1.73	(0.46, 6.48)	0.418
Bone metastases ( +/-)	2.79	(0.94, 8.28)	0.065	2.40	(0.45, 12.69)	0.304
Liver metastases (+/-)	3.83	(1.02, 14.30)	0.046	0.42	(0.03, 5.23)	0.503
OS						
Age (≥ 75/ < 74)	0.98	(0.19, 5.09)	0.977	6.72	(0.62, 73.39)	0.118
Gender (male/female)	0.85	(0.16, 4.48)	0.850	0.89	(0.16, 5.02)	0.896
ECOG performance status (2-3/0-1)	1.29 × 10^–8^	(0.00, INF)	0.999	3.32 × 10^–10^	(0.00, INF)	0.998
*EGFR* mutation status (L858R or other/exon 19 deletion)	1.75	(0.39, 7.90)	0.464	3.26	(0.81, 13.13)	0.097
PE ( +/-)	1.33	(0.30, 5.92)	0.713	0.95	(0.21, 4.32)	0.946
Brain metastases (+/-)	3.85	(0.46, 32.01)	0.213	1.77	(0.45, 7.05)	0.416
Bone metastases (+/-)	2.65	(0.51, 13.64)	0.245	3.83	(0.45, 34.19)	0.215
Liver metastases ( +/-)	3.91	(0.74, 20.72)	0.109	0.97	(0.07, 13.51)	0.982
T790M positive patients						
PFS						
Age (≥ 75/ < 74)	1.32	(0.49, 3.53)	0.577	0.88	(0.27, 2.88)	0.835
Gender (male/female)	0.52	(0.20, 1.36)	0.181	0.83	(0.26, 2.61)	0.744
ECOG performance status (2-3/0-1)	2.50	(0.94, 6.65)	0.067	1.85	(0.36, 9.65)	0.463
*EGFR* mutation status (L858R or other/exon 19 deletion)	2.09	(0.80, 5.47)	0.134	1.70	(0.46, 6.36)	0.428
PE (+/-)	4.04	(1.43, 10.92)	0.006	7.31	(2.05, 26.03)	0.002
Brain metastases (+/-)	1.50	(0.60, 3.74)	0.383	2.82	(0.71, 11.27)	0.142
Bone metastases ( +/-)	1.39	(0.55, 3.50)	0.491	0.38	(0.05, 2.65)	0.329
Liver metastases ( +/-)	1.35	(0.39, 4.70)	0.637	1.31	(0.22, 7.89)	0.766
OS						
Age (≥ 75/ < 74)	1.10	(0.35, 3.47)	0.877	1,72	(0.40, 7.49)	0.468
Gender (male/female)	0.24	(0.05, 1.05)	0.058	0.33	(0.05, 2.00)	0.226
ECOG performance status (2-3/0-1)	4.54	(1.44, 14.32)	0.010	5.12	(0.86, 30.34)	0.072
*EGFR* mutation status (L858R or other/exon 19 deletion)	1.75	(0.56, 5.51)	0.337	0.74	(0.14,4.07)	0.732
PE (+/-)	3.82	(1.34, 10.85)	0.012	4.11	(1.08, 15.62)	0.038
Brain metastases (+/-)	0.95	(0.34, 2.67)	0.920	0.72	(0.17, 3.09)	0.657
Bone metastases (+/-)	2.70	(0.95, 7.68)	0.062	1.13	(0.13, 9.80)	0.915
Liver metastases ( +/-)	3.50	(1.04, 11.73)	0.043	1.61	(0.22, 11.68)	0.640

*PFS* progression-free survival, *OS* overall survival, *HR* hazard ratio, *CI* confidence interval, *ECOG*, Eastern Cooperative Oncology Group, *EGFR* epidermal growth factor receptor, *PE* pleural effusion, *INF* infinity

## Discussion

In current study, we found that in *EGFR* T790M-negative NSCLC patients treated with osimertinib the PFS of the patients with PE were comparable to that of the patients without PE. On the other hand, the PFS and OS of *EGFR* T790M-positive patients with PE were significantly shorter in comparison to the patients without PE. The presence of PE was an independent negative predictor affecting the PFS and OS in the patients with *EGFR* T790M-positive NSCLC, but not those with *EGFR* T790M-negative NSCLC. To the best of our knowledge, this is the first report to investigate the efficacy of osimertinib in *EGFR* T790M-negative NSCLC patients with PE.

Osimertinib has been shown to be effective in untreated advanced NSCLC patients with EGFR-TKI-sensitizing mutation without T790M. The median PFS and OS of patients treated with osimertinib were reported to be 18.9 months and 38.6 months, respectively [10, 11]. The median PFS (19.8 months, 95% CI 9.0 to 25.5) in our study was consistent with that in the previous report (FLAURA study). In patients with *EGFR* T790M-positive NSCLC whose disease had progressed after EGFR-TKI treatment, the median PFS was shown to be 10.1 months [8] and the median OS was 26.8 months [9]. In our study, the median PFS (13.1 months, 95% CI 9.1 to 19.0) and the median OS (30.7 months, 95% CI 14.2 to 44.9) were similar to the results of the phase III AURA3 study [8, 9].

Previous reports showed that the efficacy of gefitinib or erlotinib were limited in patients with PE [6, 7]. In addition, Masuhiro et al. reported that similarly to first-generation EGFR-TKIs, osimertinib monotherapy appears to be less effective in patients with *EGFR* T790M-positive NSCLC with PE [12], this is consistent with our results in *EGFR* T790M-positive patients. In contrast, Kawamura et al. and Ohe et al. reported that in *EGFR* T790M-positive NSCLC treated with osimertinib, PFS did not differ to significant extent between the patients with and without PE [13, 14]. In the study reported by Kawamura et al. [13], because the patients with minimal PE (thickness < 10 mm on CT scan) were included in the group of patients without PE, the difference in PFS according PE status may be diminishing. The PFS of the patients with *EGFR* T790M mutation that were detected via malignant effusion was significantly shorter than in the patients in whom *EGFR* T790M mutation were detected by other methods [13]. In the large post-marketing study reported by Ohe et al. [14], osimertinib was effective for *EGFR* T790M-positive NSCLC, regardless of the PE status. However, the Kaplan–Meier curve for PFS in the patients with PE is slightly lower than that in the patients without PE. In this study, because the diagnosis of PE depended on the clinical judgment of the investigators, the patients with minimal PE may be included in the patients without PE. In our study, in *EGFR* T790M- positive patients, but not in *EGFR* T790M-negative patients, the PFS and OS of the patients with minimal PE were as short as those of the patients with malignant PE (Additional file 1: Figure A2, A3).

Vascular endothelial growth factor (VEGF) promotes the development of PE by increasing vascular permeability and promoting angiogenesis, and is a critical mediator in the formation of PE in lung cancer patients [18]. The serum level of VEGF was associated with the VEGF level in PE in NSCLC patients with malignant PE, and the serum level of VEGF was relatively high in the patients with malignant PE [19]. VEGF receptor 2 (VEGFR2) inhibition was reported to enhance the anti-tumor effects of EGFR-TKI in *EGFR*-mutated NSCLC models by inhibiting not only tumor angiogenesis but also oncogenic signaling in cancer cells, implying a potent role of VEGFR2 signaling in *EGFR*-mutated NSCLC cell proliferation [20]. The high level of VEGF may reduce the efficacy of EGFR-TKI in the treatment of *EGFR*-mutated NSCLC. Furthermore, the activation of EGFR signaling can upregulate the production of VEGF in human cancer cells [21], and EGFR and VEGF share a common downstream pathway, suggesting an important role of VEGF in resistance to EGFR-TKIs [22, 23]. In a preclinical study, Naumov et al. reported that EGFR-TKI resistant (primary resistant or T790M positive) cells highly secreted VEGF and that EGFR-TKI resistance could be associated with VEGF elevation in both the tumor cells and host stroma [23]. Thus, the efficacy of osimertinib for patients with PE may depend on the presence of *EGFR* T790M mutation affecting the production of VEGF.

Several clinical trials have shown that combination therapy of erlotinib plus VEGF/VEGFR blockade improves the PFS or OS in comparison to erlotinib alone in patients with *EGFR*-positive NSCLC [24–26]. However, the efficacy of EGFR-TKI combined with anti-VEGF/VEGFR antibody for patients with PE remains unclear. An exploratory subgroup analysis of the results from the JO25567 study [24] showed that PFS was significantly longer with erlotinib plus bevacizumab (*n* = 30) than with erlotinib alone (*n* = 36) in patients with pleural or pericardial effusion (15.4 vs. 5.7 months, HR 0.45, 95% CI 0.25 to 0.82) [27]. These results suggested that combination therapy with EGFR-TKI and anti-VEGF/VEGFR antibody may be a beneficial strategy for *EGFR*-mutated NSCLC with PE. A single arm phase II trial of osimertinib combined with bevacizumab for patients with *EGFR*-mutated NSCLC and malignant pleural and/or pericardial effusion is ongoing [28].

The present study was associated with some limitations. Firstly, data were obtained from a single institution and the sample size was relatively small. Thus, the difference in efficacy according to the presence of PE may have not been detected in patients with *EGFR* T790M-negative NSCLC who were treated with osimertinib. The discrepancy seen between *EGFR* T790M-positive and *EGFR* T790M-negative patients may reflect the small sample size rather than a true differential impact. Secondly, because this was a retrospective study, a collection bias may have been present. We used either an Oncomine Dx Target Test or Cobas *EGFR* Mutation Test for the *EGFR* mutations analyses at the time of the diagnosis. Many *EGFR* T790M-negative patients were diagnosed by Oncomine Dx Target Test, while almost all *EGFR* T790M-positive patients were diagnosed by Cobas *EGFR* Mutation Test. There might be difference of gene profile including uncommon mutations and compound mutations between *EGFR* T790M-positive and *EGFR* T790M-negative patients. Thirdly, the median follow-up period was 17.1 months (range, 6.9 to 31.1) for all *EGFR* T790M-negative patients. Some *EGFR* T790M-negative patients had no disease progression and were alive at the end of the follow-up period; thus the follow-up period may have been insufficient. Finally, PE has been reported to be a poor prognostic factor in patients with advanced NSCLC [1–3], PE may be a poor prognostic factor rather than a predictive factor for osimertinib.

In conclusion, this study showed that the presence of PE was a negative predictor of the efficacy of osimertinib in *EGFR* T790M-positive NSCLC patients, but not *EGFR* T790M-negative NSCLC patients. These findings suggest that among NSCLC patients with PE, the efficacy of osimertinib might differ between *EGFR* T790M-positive and *EGFR* T790M-negative patients.

## Supplementary Information

**Additional file 1.** 

## Data Availability

The datasets used and analyzed during the current study are available from the corresponding author on reasonable request.
