# Supplementary material for: Efficacy of osimertinib in epidermal growth factor receptor-mutated non-small-cell lung cancer patients with pleural effusion
Source: BMC Cancer. 2022 Jun 1;22:597. doi: 10.1186/s12885-022-09701-2 (PMC9158359; doi:10.1186/s12885-022-09701-2)
Supplement: Supplementary file 1 — Additional file 1. [file 12885_2022_9701_MOESM1_ESM.pptx]

## Slide 1
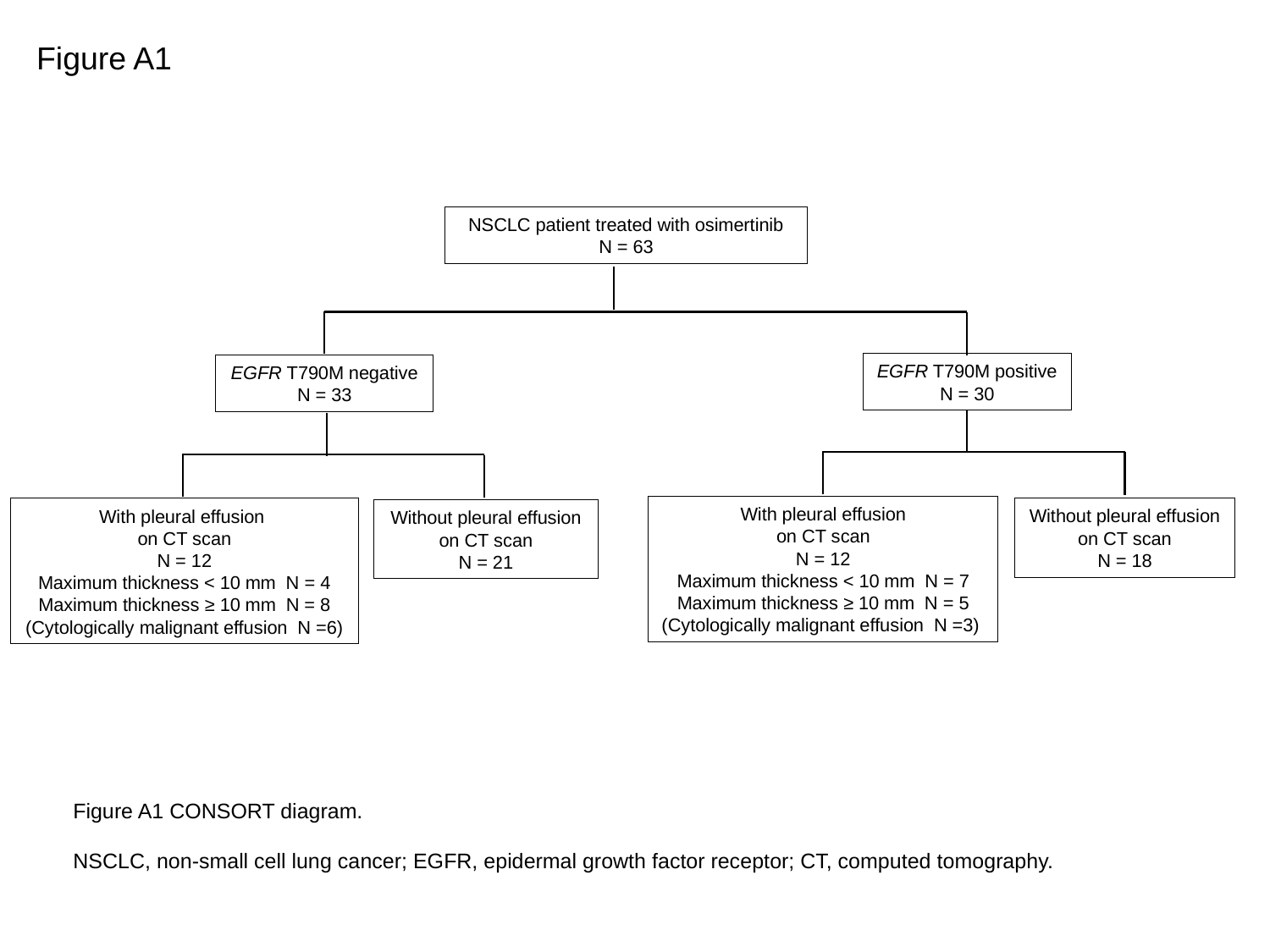

Figure A1
NSCLC patient treated with osimertinib
N = 63
EGFR T790M positive
N = 30
EGFR T790M negative
N = 33
With pleural effusion
on CT scan
N = 12
Maximum thickness < 10 mm N = 7
Maximum thickness ≥ 10 mm N = 5
(Cytologically malignant effusion N =3)
Without pleural effusion
on CT scan
N = 18
With pleural effusion
on CT scan
N = 12
Maximum thickness < 10 mm N = 4
Maximum thickness ≥ 10 mm N = 8
(Cytologically malignant effusion N =6)
Without pleural effusion on CT scan
N = 21
Figure A1 CONSORT diagram.
NSCLC, non-small cell lung cancer; EGFR, epidermal growth factor receptor; CT, computed tomography.

## Slide 2
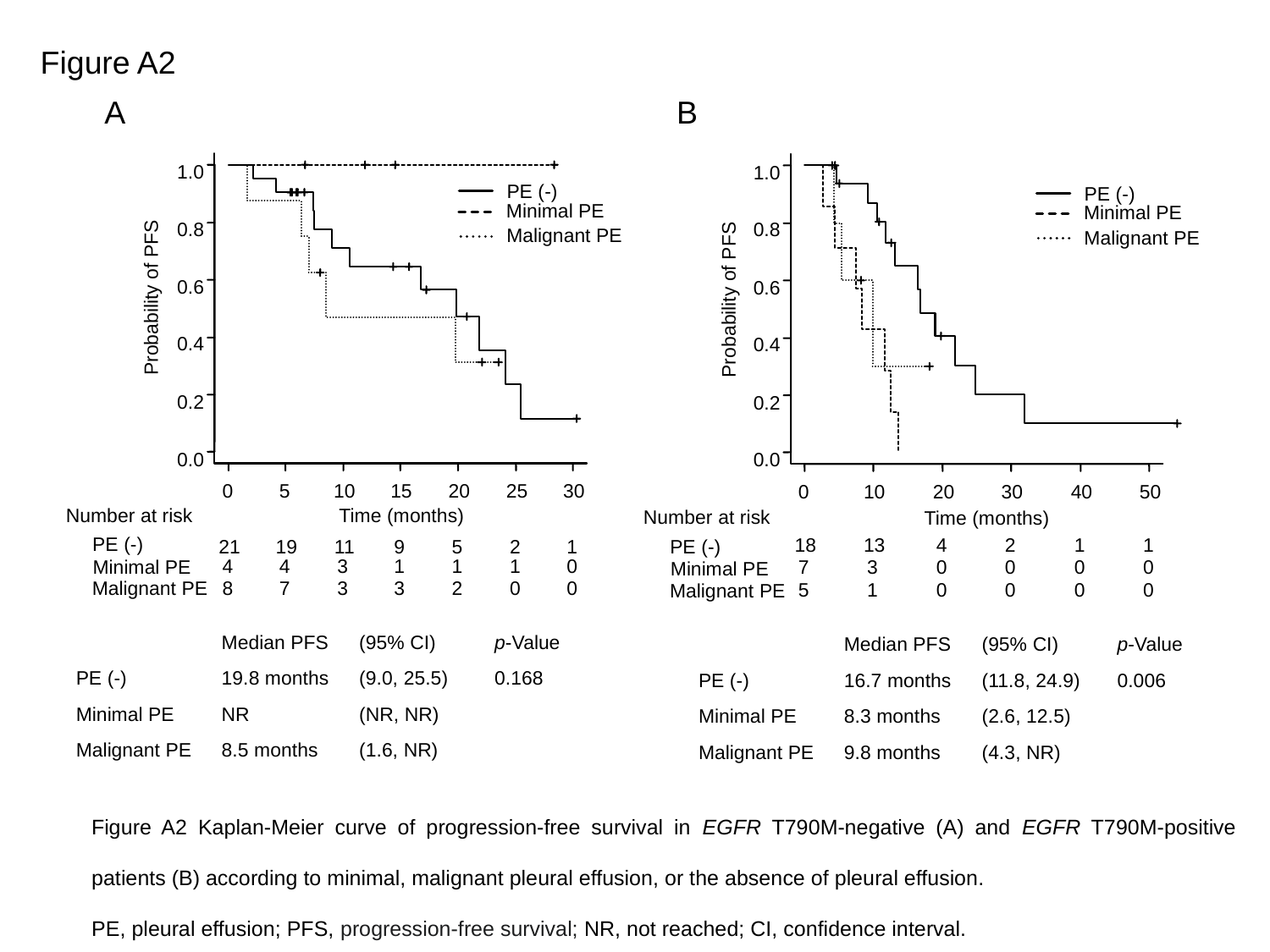

Figure A2
A
B
1.0
0.8
0.6
0.4
0.2
0.0
0
10
20
30
40
50
1.0
PE (-)
Minimal PE
Malignant PE
PE (-)
Minimal PE
Malignant PE
0.8
Probability of PFS
Number at risk
Time (months)
Probability of PFS
Number at risk
Time (months)
0.6
0.4
0.2
0.0
0
5
10
15
20
25
30
PE (-)
18
13
4
2
1
1
PE (-)
21
19
11
9
5
2
1
4
4
3
1
1
1
0
Minimal PE
7
3
0
0
0
0
Minimal PE
8
7
3
3
2
0
0
Malignant PE
5
1
0
0
0
0
Malignant PE
| | Median PFS | (95% CI) | p-Value |
| --- | --- | --- | --- |
| PE (-) | 19.8 months | (9.0, 25.5) | 0.168 |
| Minimal PE | NR | (NR, NR) | |
| Malignant PE | 8.5 months | (1.6, NR) | |
| | Median PFS | (95% CI) | p-Value |
| --- | --- | --- | --- |
| PE (-) | 16.7 months | (11.8, 24.9) | 0.006 |
| Minimal PE | 8.3 months | (2.6, 12.5) | |
| Malignant PE | 9.8 months | (4.3, NR) | |
Figure A2 Kaplan-Meier curve of progression-free survival in EGFR T790M-negative (A) and EGFR T790M-positive patients (B) according to minimal, malignant pleural effusion, or the absence of pleural effusion.
PE, pleural effusion; PFS, progression-free survival; NR, not reached; CI, confidence interval.

## Slide 3
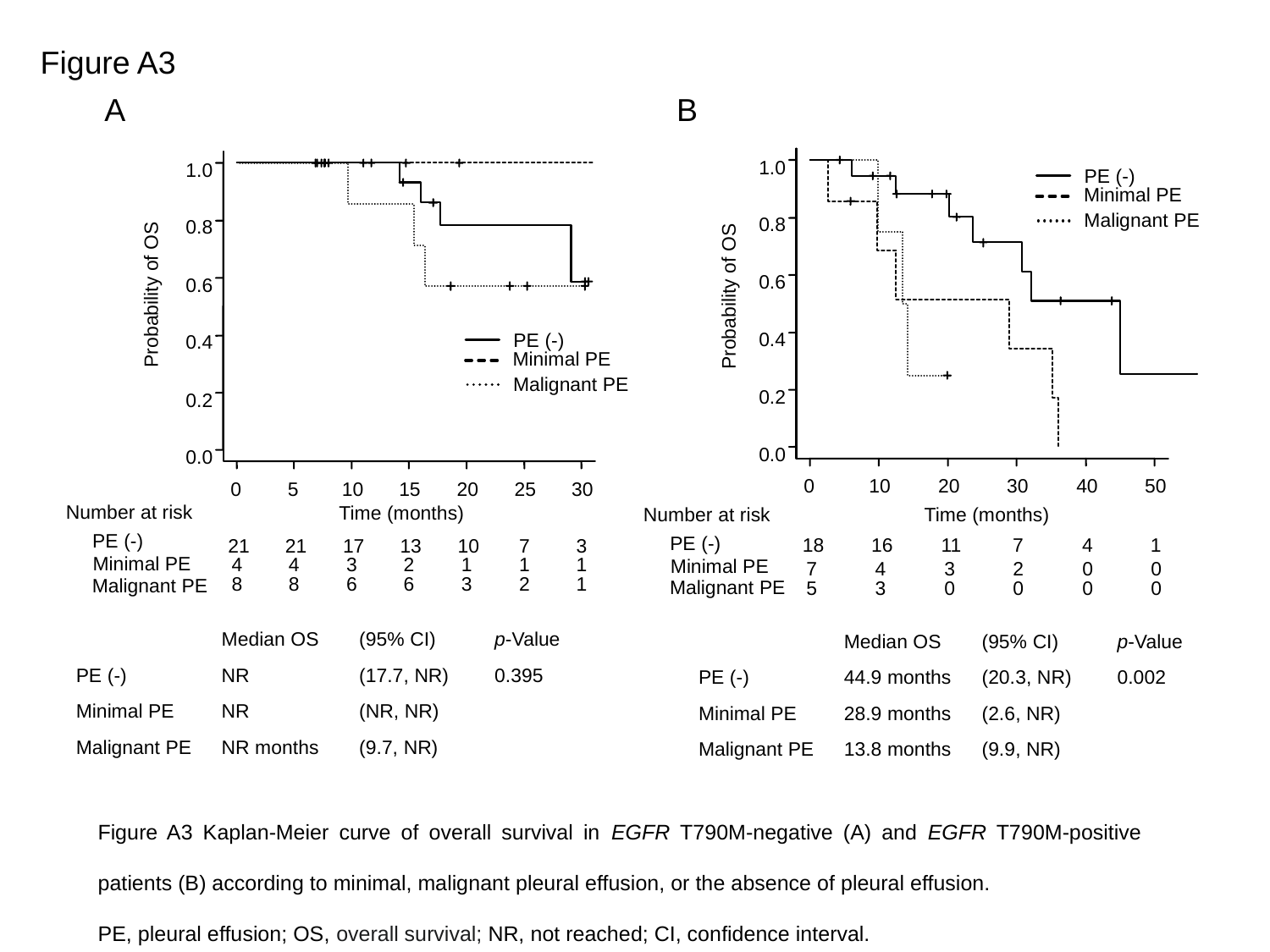

Figure A3
A
B
1.0
0.8
0.6
0.4
0.2
0.0
0
10
20
30
40
50
1.0
0.8
0.6
0.4
0.2
0.0
0
5
10
15
20
25
30
PE (-)
Minimal PE
Malignant PE
Probability of OS
Number at risk
Time (months)
Probability of OS
Number at risk
Time (months)
PE (-)
Minimal PE
Malignant PE
PE (-)
PE (-)
18
16
11
7
4
1
21
21
17
13
10
7
3
Minimal PE
4
4
3
2
1
1
1
Minimal PE
7
4
3
2
0
0
8
8
6
6
3
2
1
Malignant PE
Malignant PE
5
3
0
0
0
0
| | Median OS | (95% CI) | p-Value |
| --- | --- | --- | --- |
| PE (-) | NR | (17.7, NR) | 0.395 |
| Minimal PE | NR | (NR, NR) | |
| Malignant PE | NR months | (9.7, NR) | |
| | Median OS | (95% CI) | p-Value |
| --- | --- | --- | --- |
| PE (-) | 44.9 months | (20.3, NR) | 0.002 |
| Minimal PE | 28.9 months | (2.6, NR) | |
| Malignant PE | 13.8 months | (9.9, NR) | |
Figure A3 Kaplan-Meier curve of overall survival in EGFR T790M-negative (A) and EGFR T790M-positive patients (B) according to minimal, malignant pleural effusion, or the absence of pleural effusion.
PE, pleural effusion; OS, overall survival; NR, not reached; CI, confidence interval.
